# Challenges to the utilization of Community-based Health Planning and Services: the views of stakeholders in Yendi Municipality, Ghana

**DOI:** 10.1186/s12913-021-07249-8

**Published:** 2021-11-11

**Authors:** Bougangue Bassoumah, Andani Mohammed Adam, Martin Nyaaba Adokiya

**Affiliations:** 1 Department of Population & Reproductive Health, School of Public Health (SPH), University for Development Studies (UDS), Tamale, Ghana; 2 Faculty of Social Sciences, Universiti Malaysia, Sarawak Kota Samarahan, Malaysia; 3 Department of Epidemiology, Biostatistics & Disease Control, SPH, UDS, Tamale, Ghana

**Keywords:** Community-based Health Planning and Services, Health inequality, Implementation, Challenges, Clinic attendance

## Abstract

**Background:**

The Community-based Health Planning and Services (CHPS) is a national health reform programme that provides healthcare at the doorsteps of rural community members, particularly, women and children. It seeks to reduce health inequalities and promote equity of health outcomes. The study explored implementation and utilization challenges of the CHPS programme in the Northern Region of Ghana.

**Methods:**

This was an observational study that employed qualitative methods to interview key informants covering relevant stakeholders. The study was guided by the systems theory. In all, 30 in-depth interviews were conducted involving 8 community health officers, 8 community volunteers, and 14 women receiving postnatal care in four (4) CHPS zones in the Yendi Municipality. The data were thematically analysed using Atlas.ti.v.7 software and manual coding system.

**Results:**

The participants reported poor clinical attendance including delays in seeking health care, low antenatal and postnatal care visits. The barriers of the CHPS utilization include lack of transportation, poor road network, cultural beliefs (e.g. taboos of certain foods), proof of women’s faithfulness to their husbands and absence of health workers. Other challenges were poor communication networks during emergencies, and inaccessibility of ambulance service. In seeking health care, insured members of the national health insurance scheme (NHIS) still pay for services that are covered by the NHIS. We found that the CHPS compounds lack the capacity to sterilize some of their equipment, lack of incentives for Community Health Officers and Community Health Volunteers and inadequate infrastructures such as potable water and electricity. The study also observed poor coordination of interventions, inadequate equipment and poor community engagement as setbacks to the progress of the CHPS policy.

**Conclusions:**

Clinical attendance, timing and number of antenatal and postnatal care visits, remain major concerns for the CHPS programme in the study setting. The CHPS barriers include transportation, poor road network, cost of referrals, cultural beliefs, inadequate equipment, lack of incentives and poor community engagement. There is an urgent need to address these challenges to improve the utilization of CHPS compounds and to contribute to achieving the sustainable development goals.

## Introduction

The Community-based Health Planning and Services (CHPS) is one of the prioritized policies to promote safe motherhood in rural Ghana [1]. The CHPS provides a range of services including antenatal care (ANC), postnatal care (PNC) through Community Health Officers (CHOs), and emergency delivery during the crowning point of labour and delivery [2]. This programme uses community health volunteers (CHVs) who are supported by communities, including the recruitment, training, and deployment of volunteer workers to provide family planning services and refer clients to CHOs [3].

CHPS is a national health reform that mobilizes volunteerism, resources, and traditional institutions to support community-based primary healthcare. It is implemented through the provision of healthcare at the doorsteps of community members, particularly, rural women and children. The CHPS programme enables the Ghana Health Service (GHS) to reduce health inequalities and promote equity of health outcomes [2, 3]. The implementation of the CHPS needs the co-operation of the health sector and the communities in which the programme is being run. The CHPS embraces systematic planning and negotiation with all stakeholders; local authorities, political leaders, and community members through community mobilization and effective participation [3].

In general, there is improvement in ANC, skilled delivery (SD), and PNC. However, the progress does not cover the whole country due to regional or locational inequalities in access to health information and services [4, 5]. This uneven progress has resulted in widening inequalities in health outcomes [5]. Despite the improvement in SD, only one-third of pregnant women in Ghana who need emergency care do receive it. Thus, widening the regional inequality gap [6]. The CHPS intervention has been subjected to experiment and proven that engaging a resident nurse in a community and involving traditional leaders and community members in the provision and management of healthcare substantially ensures male participation and improves maternal and child health outcomes as well as health system accountability and strengthening [5]. Some of the challenges include cultural factors, lack of professionalism, poor clinical setup, and long-distance with an associated cost. These key factors influence women’s choice of health facility in maternity [7]. Skilled delivery care is essential in improving child and maternal mortality and morbidity in developing countries [5]. Though the CHPS strategy is a prioritized intervention to improve maternal and child health [1], the provision of SD care is not stated as a core activity in the policy [8, 9]. The initiative aims at promoting SD care through community participation, promotional activities, and referral services [8, 10], with emphasis on expansion of CHPS zones to address geographical access, and remove financial and cultural barriers to clinical care [6].

Most of the existing studies focused on assessing the nation-wide performance of the CHPS initiative. This paper was motivated by the observation of increasing concerns of widening inequalities at the sub-national level [11]. There is evidence that in situations where national maternal and newborn mortality rates reduced, there are sub-groups where survival rates and access to services have not changed or rather worsened over time [12–15, 5]. Further, there have been reports on poor clinical attendance. This is associated with high maternal and neonatal morbidities and mortalities in the northern region of Ghana [16]. In this study, we explored the implementation challenges of the CHPS intervention from the views of women, CHVs, and CHOs. Given the urgency to accelerate the progress of the Sustainable Development Goal 5 (Achieving Gender Equality and Empowering Women and Girls towards 2030), this study is essential for informing health policy development and practice for the improvement and sustenance of the well-being of women and their dependents.

### Theoretical framework

The conceptual framework of this study was founded on the social ecological model [17, 18]. The social ecological model is a theory-based framework for understanding the multifaceted and interactive effects of dimensions such as individual (e.g. knowledge, socioeconomic status, religious affiliation, financial resources), interpersonal (e.g. families, friends, customs or traditions), community (e.g. community leaders, transportation, village associations), and policy (e.g. policies regarding the allocation of resources for maternal, newborn, and child health, access to healthcare services) on human behaviour. In other words, this framework can be used to demonstrate how challenges in the utilization of CHPS result from a complex interaction of individual, relationship, community, and social factors.

## Methods

### Study setting

The Yendi Municipality is located in the Eastern corridor of the Northern Region. The Municipality shares boundaries with six (6) other District Assemblies; to the East; Saboba District, Chereponi District and Zabzugu District, to the South Nanumba North District, to the North Gushegu District and Mion District to the West. The Municipality is strategically located at the center of the Eastern Corridor of the northern, with a landmass of 1,446.3 sq km. [16]. It is about 90 km from the Northern Regional capital, Tamale. According to the 2010 Population and Housing census, the population of the Municipality is estimated at 117,780 and has varied ethnic groups with the Dagomba constituting the majority. The other ethnic groups include Konkomba, Akan, Ewe, Basare, Moshie, Chokosi and Hausa. The population is largely rural with 56 % of the population living in rural areas. Out of the total population, 50 % are males. The main religious groupings are Moslems (67.2 %), Christians (17.4 %), Traditionalist (13.2 %), No Religion (1.8 %) and other (0.3 %). In terms of health infrastructure, the population is served by one public hospital located in Yendi, four health centers, two clinics, one of which is privately owned, and four Community-based Health and Planning Services (CHPS) compounds. The human resource situation in the municipality is not the best; some of the health facilities are being managed with inadequate staff [4].

### Study design

 This was a cross-sectional explorative study involving community health officers (CHOs), community health volunteers (CHVs) and women receiving postnatal care in selected CHPS facilities in the Yendi Municipality in Northern Region of Ghana.

### Sample and sampling approach

The participants were selected using a variety of non-probability sampling techniques. Firstly, the researchers categorize the sub-districts into urban and rural. All the CHPS facilities in each sub-district in the Municipality were included in the study. The team observed that the selected CHPS had only females as CHOs, Therefore, the CHOs in the present study were all females. Two CHOs were interviewed from each CHPS facility. Furthermore, two CHVs were purposively selected from each CHPS zone. Regarding the selection of PNC women for the study, the research team informed a gatekeeper (CHV) about the characteristics of the PNC women required for the study. The quota technique was used to ensure equitable distribution of participants according to some key socio-demographic characteristics such as age and location. The educational level of the women was also taken into consideration in the selection. Overall, 4 CHPS, 8 CHOs, 8 CHVs, and 14 PNC women were selected to participate in this study.

### Data collection

Three data collectors with at least a diploma qualification and experience with qualitative data collection were recruited to help with the data collection process. The data collectors were also proficient in English and Dagbani. A member of the research team organized a one-day training session for the data collectors to improve their interviewing skills. Lectures, demonstrations, and practice were used to achieve the objectives of the training session. Interview guides were used to collect information for the qualitative study. The interview guide was pretested among a group of participants, including 4 CHVs, 4 CHOs, and 7 PNC mothers from a neighbouring district (Saboba district to the north east, mion district to the West, Tatale district to east, Zabzugu to east and Nanumaba North to the south of Yendi) to ensure validity and reliability of guides in collecting the desired information. Ambiguous questions were modified accordingly. The interviews were conducted in either the English Language or Dagbani, depending on the respondent’s preferred language. However, interviews with CHOs were conducted in English and took place at the CHPS facility while the CHVs and PNC women were interviewed in their respective homes in either English or Dagbani. All the interviews were tape recorded to capture the responses of the participants. Each interview lasted an average of 45 min.

Some of the questions asked during the data collection were; 1). As pregnant women/mothers, what challenges do you face in using the CHPS compound in your community? Are you satisfied with the services provided by CHOs in your CHPS compound? If yes/no, could you give reasons for your answer? 2). Having been involved in volunteerism towards the progress of the CHPS compound, what do see as implementation challenges to the programme? What problems do you face as volunteers in rendering your services? What do you think should be done to enable you to do your work well for the success of the CHPS programme? 3). Do the women in this area patronize the services of this CHPS compound? If no, why do you think they are not using the facility? What are the problems you face as CHOs in rendering your services?

### Data analysis

In this study, two research assistants transcribed the recordings verbatim (FKC and GNA). The third author (MNA) who has extensive experience in transcription validated the transcripts from the interviews conducted in Dagbani together with one research assistant (GP), who was proficient in Dagbani for accuracy of the translation into the English language. We imported the transcripts into Atlas.ti.v.7 software for the final analysis. Braun and Clark’s thematic analysis approach was used to analyze the data (Braun & Clarke, 2006). The thematic analysis technique is simple to implement and provides a thorough analysis of the data generated. We ensured that there was sufficient data to support each potential theme. In brief, the analysis began with reading the transcripts repeatedly in order to understand the data, identify overall themes, and summarize the responses to each vignette. We also performed open coding by highlighting sections of the text that were relevant to the study objective, emerging themes and sub-themes. Then, to establish similarities between themes, axial coding was used. We made certain that there was sufficient data to support each theme. As a result, some themes were collapsed, renamed, or even deleted because they were unifying. We paraphrased respondents’ responses and used quotations where appropriate. The steps in the thematic analysis have been included as an additional file in this manuscript.

### Data quality and control

Threats to the validity and reliability of the data collected were addressed using the following strategies. Firstly, the interview guide was shared with experts in qualitative research methodology from the University for Development Studies for evaluation and modification. Secondly, to ensure that participants understood the questions as intended, the interview guide was translated from English to Dagbani and back to English using the translate-back-translate method. Thirdly, before collecting data, the interview guide was pretested. The pre-test ensured the validity and reliability of the data collection tool in gathering the needed information. The data gathered from the pre-test was excluded in the final analysis. Fourthly, data collectors with at least a diploma in the level of education and with previous experience(s) in gathering qualitative data were used for the data collection in the study. A one-day face-to-face training was also organized for the data collectors, who were also supervised throughout the data collection period. Lastly, the interviews were audio-recorded and verbatim transcribed to reflect the opinions of the participants.

## Results

### Socio-demographic characteristics of key informants

The majority of the CHOs (*n*=5) and PNC women (*n*=6) belonged to the age group of 20-34 years while all the CHVs were 35 years or older. Regarding the educational level of the participants, 6 CHVs had JHS level education whereas 8 PNC women had JHS level education (Table 1).

**Table 1:** Socio-demographic characteristics of key informants

Characteristic	Category of respondent		
	Community Health Officer	Community Health Volunteer	Postnatal Care women
Age (Years)			
<20	0	0	4
20-34	5	0	6
≥ 35	3	8	4
Education			
No formal education	0	0	4
Primary	0	1	0
Junior High School	0	6	8
Senior High School/Vocational	0	1	2
Certificate	6	0	0
Diploma	2	0	0

### CHPS utilization access challenges

The participants reported that transportation was a barrier to the utilization of the CHPS facilities. Thus, a mother’s ability to use a motorbike is likely to facilitate the utilization of the CHPS compounds. For mothers who had access to or owned motorbikes, heavy rains and poor road networks were cited as challenges affecting utilization of the CHPS compounds. Some participants also reported the absence of the CHOs at the health facility.

*“Though I have a motorbike, I cannot even use it during the rainy season due to the nature of the road linking this community to the health facility. I may not be fortunate to meet CHO who can solve my problem. Sometimes, we are asked to go to Yendi Hospital when the health problem is complicated or absence of CHO” ***(Mother, 27 years).**

For instance, some remote settlements have no access roads and means of transport to facilitate attendance to CHPS compounds. They reported that the ambulance service and communication network are poor. Some communities with access to good roads faced the problem of means of transport, with a worse situation for women in remote areas. In the study setting, it was reported that there are few commercial vehicles running in some areas.

*“You can see for yourself. The road linking this village to the next community with a CHPS facility is a footpath. When I was about to deliver, I was carried on a bicycle to the facility. The pain was too much I opted for walking but the distance to the facility was far. On the way, we had to branch to the house of a TBA for delivery” (***Mother, 22 years***)”*.

This implies that one’s ability to afford transportation and the cost associated with clinical care may not lead to access to health care. But the nature of roads and lack of expertise in the CHPS compound may prevent access and utilization. Similarly, referrals to Municipal level health facilities may pose other challenges which lead to poor utilization of health care.

### Poor coordination of interventions

In this study, we found that the maternal healthcare policy interventions such as the national ambulance service, safe motherhood protocol, national health insurance scheme, and medical supplies and health personnel are not well coordinated at the implementation level. These policies are developed and rolled-out without proper consideration of the implementation phase. They also reported of limited communication network which makes it difficult for women or their families to contact private transport operators, ambulance service providers, and the CHOs during emergencies. Apart from the municipal hospital which is a referral centre, none of the CHPS compounds has an ambulance for emergencies and referrals..

*“… We do not have an ambulance for emergencies. Only the district hospital has it. Hmm, … we are trying our best to save our fellow women during complications and emergencies.“***(Community Health Officer***).*

### Delays in seeking care

The participants reported delays at the household and community levels due to cultural beliefs. At the health facility level, the safe motherhood protocol that is supposed to give priority to maternity cases was not functioning well. The majority of the women admitted to delays in seeking care due to poor clinical setup, the attitude of some professionals, and understaffing:

*“When I got there* (CHPS compound), *… I further waited for close to an hour before a good person picked me and transported me to the hospital. I cried for 30 minutes and nobody came to me until I reached the crowning stage … After delivery, they told me that the midwife and the doctor are engaged” (***Mother, 35 years***)*.

We also identified variation in policy and implementation practice between the National Health Insurance Scheme (NHIS) and safe motherhood protocol in the provision of medical services, particularly in the referral facilities. The majority of women reported having to pay unauthorized money for services that are covered by NHIS.

*“I have an NHIS card but I was made to pay for a laboratory test. Is it not cheating? I do not know why they do not do what they promise in the NHIS policy.“ (***Mother, 38 years***)*.

When further inquired, we found that some of the health facility-based delays connected to the provision of medicines had to do with the procurement law that restricted the process of acquisition of medical supplies. Some of the CHPS compounds are like the traditional birthing settings without any standardized equipment or materials to facilitate healthcare delivery. The services do not meet the healthcare needs of clients. For instance, they had no sterilizers and depended on the district hospital for such service. However, the women were comfortable with the CHOs in their communities compared to professionals in referral facilities due to familiarity with the setting.

*“We always blame people for not using CHPS compounds, but sometimes they are justified. Tell me, is this place a health facility or a bedroom?“ (***Community Health Officer***).*

*“One of our challenges is the lack of equipment and logistics. Sometimes, we do not have the basic items required for safe delivery. The mothers are asked to bring soap, disinfectants, and gloves. Even to sterilize some of the equipment, we have to send it to Yendi Municipal Hospital”* (**Community Health Officer**).

*“I enjoy being assisted at delivery by CHOs in this community compared to the midwives in the municipal hospital. The CHOs’ behaviour is closer to that of a TBA. They show love to us and they have understanding.“* (**Mother, 28 years**).

### Cultural beliefs and practices

The participants reported that beliefs around pregnancy and childbirth are one major challenge to receiving CHPS services. This results in a dualism of care-seeking, which leads to poor clinical attendance, delays in seeking healthcare, and a low number of ANC visits. The health beliefs and practices were repeatedly reported as CHPS programme challenges by participants.

*“I made two visits to the CHPS compound but when it was time for delivery, I delivered at home. Oh! I used medicines from the health centre alongside herbs from my mother in-law” (***Mother, 34 years***).*

Due to fear of referrals from CHPS facilities to Yendi Municipal Hospital, some women avoided the CHPS services. The main reason for avoidance of referrals to intensive care is to ensure that tabooed foods are not received but prefer to receive treatment from the traditional practitioners based on their beliefs about the causes of pregnancy-related complications. Similarly, women who seek to prove their faithfulness and fidelity to the marriage, are encouraged to have home delivery. So that when they have prolonged labor or complications, the women may be forced to give confession to determine the paternity of the pregnancy. Also, women who received treatment from traditional practitioners before the pregnancy are made to deliver in their custody for rituals to be performed.

*“When we make referrals, some of the women refuse the referral services for fear of caesarean section. They prefer to receive herbal treatment or to avoid forbidden food that may be provided at the referral health facilities.“ (***Community Health Officer***)*.

*“When I got pregnant, my husband told me that it was my former boyfriend who was responsible for the pregnancy. This generated a misunderstanding between us. So, he neglected me. When I was in labour, I decided to go for skilled delivery but my husband refused and invited a traditional birth attendant to the house to assist at delivery to make me confess in the event of prolonged labour or placenta retention. … but I gave birth easily.“***(Mother, 40 years).**

*“Both my husband and the herbalist insisted that I should give birth in the custody of the herbalist for necessary rituals to be performed before, during, and after delivery.“ (***Mother, 30 years).**

### Lack of incentives

The community health volunteers (CHVs) and community health officers (CHOs) who participated in this study complained of a lack of incentives to motivate their activities. The CHVs were married men and women with family responsibilities. The need to cater to their families did not allow them to give proper attention and commitment to the volunteer activities. While the CHOs had problems with housing, potable water, and reliable electricity which hindered their activities. As workers in deprived communities, they did not have any incentives as motivation.

*“We are doing voluntary work but people think we are paid. We leave our farms and spend time on this voluntary service, but we do not even get common thank you.“ (***CHV, aged 46***).*

*“I have been working at this facility for the past 3 years. We do not have proper housing here and the materials needed to facilitate our work are also lacking. For electricity and water, the least said about them the better” (***Community Health Officer).**

### Lack of specialists and equipment

Health facilities are required to be equipped with standardized items for maternal and child healthcare. We found that the CHPS compounds did not have adequate human and material resources for effective and efficient healthcare delivery. For instance, none of the four (4) health facilities had laboratory equipment and specialists to deal with pregnancy-related complications. Though these services are provided in the municipal hospital, the poor road network and non-availability of ambulances and other means of transport as well as the cost involved making it difficult to utilize healthcare services for a healthier life. Thus, mothers who cannot afford referral costs ignored skilled care and resorted to traditional birth attendants (TBAs) and herbalists. According to the CHOs, certain services are beyond the mandate of the CHPS compounds. Thus, CHOs only provide services when they are mandated and provided with human and material resources.

“*Some of the health workers are very good, but some women and their husbands would rather go for TBAs’ services in the event of serious complications for nature to decide. Those who can afford are asked to go to Yendi Hospital for expert care. Because in this village no matter what they will ask you to go to Yendi for laboratory tests or to see a doctor.“***(Male Community Health Volunteer, 45 years).**

*“Yes, we are limited in providing healthcare. So we give first aid in some instances and refer patients to specialists at Yendi. Probably due to the cost involved, most of the women do no go.“***(Community Health Officer, 24 years).**

### Poor community engagement

We also found that the engagement of community members particularly men in the CHPS programme as outlined in the policy is very poor. The CHVs and women reported that they hardly met with the staff of Ghana Health Service for the CHPS initiative development programmes.

*“When they established this CHPS compound, they promised us regular programmes and meetings but they do not come. So they expect us to stop our work and follow something that they do not value themselves?“ (***CHV, aged 34***).*

The CHVs and mothers reported that the CHOs are duty conscious and have good working relationships with community members. They worked closely with the TBAs to facilitate clinical attendance of pregnant and newborn mothers and referrals of cases beyond their capability to the municipal hospital.

*“For the two times I visited the CHPS compound, I realised that the health workers here are hardworking and friendly. They relate well with us the women especially when we come for clinical care. They normally go round to talk to pregnant women specially to come for check-up … Yes, they respect people well.“***(Mother, 28 years).**

## Discussion

This study used a qualitative approach and the social ecological model to explore the challenges to the utilization of CHPS in the Yendi Municipality of the northern region of Ghana. The analysis identified community and the health system-related factors, which influence the use of services from CHPS facilities. These factors act either independently or in combination.

Irrespective of other background variables, the data showed a poorer attendance for women from remote communities without CHPS compounds as compared with their counterparts who had CHPS compounds in their communities. This was attributed to transportation challenges. The provision of healthcare facilities without corresponding provision of transport or ambulance services means women’s movement to seek maternity services will be restricted [19, 20]. The various actors of the healthcare system should consider the inter-connectedness of NHIS, safe motherhood protocol, regular supply of equipment/medicines as well as the availability of qualified medical personnel for positive outcomes. These are important components of the healthcare system that should work together to make progress because their functions or effects have a cumulative effect on the outcome of the CHPS policy. Therefore, when any of these is/are not functioning well, the provision of healthcare services will not meet the expected outcome.

Another key issue worth noting is that the CHPS compounds were not equipped enough with the required facilities, equipment, supplies, and human resources to meet the maternal health needs of the communities. They were not in the best position to provide Skilled Delivery (SD) care and emergency obstetric care (EoC). Though these services are not part of the core operational duties of the CHPS initiative, being the first point of contact in the healthcare system’s structure, CHPS compounds need to be equipped and empowered to provide such services if maternal health is to be improved.

The National Health Insurance Scheme (NHIS) was initiated to address the financial challenges [21] and CHPS was to make basic services available at the doorsteps of clients [22]. However, findings from this study concur with previous studies [23, 24] that the introduction of NHIS has generated a collection of unauthorized fees in the facilities from policyholders for services covered by the policy. Again, disbursement of NHIS claims and bureaucracy in procurement were key challenges to professionals in the acquisition of medical supplies. The affordability of healthcare services by the women was through the use of NHIS cards or cash payment for the non-insured. However, particularly in the referral facilities, some policyholders had no access to care for services covered by NHIS unless they paid for such services. This rather gave wider coverage of services to the rich which conflicts with the aim of the policy and synonymous with the cash and carry system that NHIS replaced. This sustains previous study findings in developing countries that the introduction of health insurance with free maternity care has resulted in the collection of unofficial fees in health institutions [12, 23]. Procurement difficulties were observed to be the cause of delays in the provision of medical supplies which resulted in artificial shortages. This forced the professionals in some instances to ask clients to purchase medicines from private pharmacy shops. Procurement is one of the most cited healthcare barriers accounting for 65 % in developing countries [25]. This calls for a re-examination of the procurement law for the healthcare system to remove the associated barriers for timely skilled therapy to be effected.

It was evident that women in remote areas continue to give birth at home without assistance from the CHOs, which sustains the argument that it is increased but inequitable access to CHPS services to the disadvantage of remote communities without CHPS compounds [26, 5]. The cordial relationship between CHOs and CHVs enhanced clinical services utilization by women in the CHPS compounds and the nearby conventional facilities. The CHOs worked closely with the CHVs to actively provide SD care through referrals and encouraging women to receive skilled care at the conventional facilities. Skilled care has the potential of reducing neonatal mortality by 25 % and the provision of basic emergency obstetric care and comprehensive neonatal care can reduce these deaths by 40 % and 85 %, respectively [9]. However, the CHPS initiative was limited in operation because they lacked expertise and did not have the mandate by the provision of the policy to offer such services. To improve and sustain the well-being of rural women, the CHPS policy needs revisitation to include all components of maternity care and deploy experienced or well-equipped personnel to the CHPS compounds. Previous research has identified human resource challenges in CHPS zones as a key factor that explains the low uptake of skilled maternity care in marginalised rural communities [6, 27]. This means quality and uptake of skilled care could be improved significantly in CHPS zones with skilled midwives [28]. The challenges with the transport system and the national ambulance service call for the use of properly trained CHOs to provide SD care within the communities they serve, particularly where access to health facilities is constrained.

A neglected and essential influence of maternal healthcare services utilisation is the power of culture [19, 30]. The CHPS policy recognises cultural beliefs and practices, and actively engages communities particularly traditional authorities in community sensitization programmes. However, the findings show that this has not received comprehensive attention at the implementation level. Culture is the foundation of every society and behavioural changes must take place through culture [31]. For the modern healthcare system to be accepted and received, the perpetrators of culture, particularly men should be part of the change process and be accountable for the outcomes. Community engagement and participation are critical in facilitating a sustainable primary healthcare system [3]. The inclusion of traditional leaders in CHPS implementation will promote acceptance and sustainable use of orthodox healthcare services [32]. It is an essential strategy to educate people of different backgrounds through needs assessment on the causes and effects of some medical conditions that require professional intervention [22].

Workers in deprived communities should be motivated with incentives such as decent housing, electricity, and potable water supply. It is a commitment to accept postings to rural areas where most basic life necessities are lacking. The recommendation for improving housing conditions for CHOs since 2011 is yet to be implemented in many CHPS zones in the region [22, 33]. Over the years, effort in developing volunteers has not been as consistent as that in developing the CHO component of the strategy [22]. This is partly because the CHVs are not motivated enough for people to be attracted and committed to volunteerism, which is an additional responsibility apart from their economic activities.

### Limitations of the study

The study protocol was only reviewed by a committee responsible for evaluating postgraduates’ proposals at the Faculty of Social Sciences and Humanities, and the School of Graduate Studies, Universiti Malaysia Sarawak, Malaysia, unfortunately, approval was given without an ID.

## Conclusions

Though a promising initiative, the study discovered a lack of a comprehensive approach in the implementation of CHPS policy. The study indicated that implementation of the CHPS policy did not give particular attention to the effects of the functions of the individual components or actors of the healthcare system and their cumulative effects on the CHPS policy outcome. It needs to be highlighted that the implementation of CHPS policy needs to be approached with holistic and systemic lenses. The initiative, therefore, requires proper coordination of all actors and emphasis on the interrelatedness of other policy interventions of the healthcare system to bridge the equity gap in access to healthcare regardless of the location and socio-economic backgrounds of women.

The Ministry of Health and the Ghana Health Service should consider the cumulative effect of other healthcare actors and interventions in health promotion planning and implementation programmes. Thus, the achievement of the Sustainable Development Goal of improvement and sustenance of women’s well-being by 2030 requires re-visitation and reformation of the CHPS policy by looking at the interconnectedness of all aspects of the healthcare system for equilibrium to be established. This would make healthcare services more accessible and acceptable to the women and their families for positive maternal outcomes.

## Data Availability

To ensure confidentiality, and as the topic is culturally sensitive, the raw data will not be deposited in publicly available repositories. However, data can be obtained from the corresponding author on reasonable request.

## Abbreviations

ANC: Antenatal Care
CHOs: Community Health Officers
CHPS: Community-based Health Planning and Services
CHVs: Community Health Volunteers
EoC: Emergency Obstetric Complication
GHS: Ghana Health Service
GSS: Ghana Statistical Service
GoG: Government of Ghana
MoH: Ministry of Health
NHIS: National Health Insurance Scheme
TBA: Traditional Birth Attendant
PNC: Postnatal Care
SD: Skilled Delivery
SDS: Sustainable Development Goal
UN: United Nations
